# Developmentally regulated long non-coding RNAs in *Xenopus tropicalis*

**DOI:** 10.1016/j.ydbio.2016.06.016

**Published:** 2017-06-15

**Authors:** Elmira Forouzmand, Nick D.L. Owens, Ira L. Blitz, Kitt D. Paraiso, Mustafa K. Khokha, Michael J. Gilchrist, Xiaohui Xie, Ken W.Y. Cho

**Affiliations:** a Department of Computer Science, University of California, Irvine, CA 92697, USA; b The Francis Crick Institute, Mill Hill Laboratory, The Ridgeway Mill Hill, London NW7 1AA, UK; c Department of Developmental and Cell Biology, University of California, Irvine, CA 92697, USA; d Program in Vertebrate Developmental Biology, Department of Pediatrics and Department of Genetics, Yale University School of Medicine, 333 Cedar Street, New Haven, CT 06520, USA

**Keywords:** *Xenopus tropicalis*, Non-coding RNA, Expression profile, RNA-seq, Gene expression, Systems biology, Gaussian processes, Biological timescales

## Abstract

Advances in RNA sequencing technologies have led to the surprising discovery that a vast number of transcripts emanate from regions of the genome that are not part of coding genes. Although some of the smaller ncRNAs such as microRNAs have well-characterized functions, the majority of long ncRNA (lncRNA) functions remain poorly understood. Understanding the significance of lncRNAs is an important challenge facing biology today. A powerful approach to uncovering the function of lncRNAs is to explore temporal and spatial expression profiling. This may be particularly useful for classes of lncRNAs that have developmentally important roles as the expression of such lncRNAs will be expected to be both spatially and temporally regulated during development. Here, we take advantage of our ultra-high frequency (temporal) sampling of *Xenopus* embryos to analyze gene expression trajectories of lncRNA transcripts over the first 3 days of development. We computationally identify 5689 potential single- and multi-exon lncRNAs. These lncRNAs demonstrate clear dynamic expression patterns. A subset of them displays highly correlative temporal expression profiles with respect to those of the neighboring genes. We also identified spatially localized lncRNAs in the gastrula stage embryo. These results suggest that lncRNAs have regulatory roles during early embryonic development.

## Highlights

• Community resource: a catalog of developmentally regulated long non-coding RNAs for *Xenopus tropicalis.*
• Temporal and spatial expression profiling of multi-exon and single-exon lncRNAs over the first 3 days of development.
• Expression correlation between lncRNA and neighboring genes.

## 1 Introduction

Advances in RNA sequencing technologies have identified a large cohort of ncRNA species that have distinct functions (Rinn and Chang, 2012), which can be subdivided into two groups. Short ncRNAs (sncRNAs) include microRNAs (miRNAs), short interfering RNAs (siRNAs) and piwi-interacting RNAs (piRNAs). In contrast, long ncRNAs (lncRNAs) are considered to be greater than 200 nucleotides in length, transcribed by RNA polymerase II, and usually polyadenylated (Ulitsky and Bartel, 2013). LncRNA loci are also characterized by having epigenetic markers typical of protein coding genes (Prensner and Chinnaiyan, 2011). A systematic annotation of lncRNA genes is not available for most organisms, and even in those with such annotation, only a small percentage of known lnRNAs have been subject to in depth experimental study to ascertain their functions. A few well-known lncRNAs are *Xist, H19,* and *HOTAIR.* The *Xist* gene is involved in silencing of the X-chromosome (Brown et al., 1991, Gendrel and Heard, 2014). *H19* brings repressive histone marks to the differentially methylated regions of target genes (Bartolomei et al., 1991). *HOTAIR* interacts with Polycomb repressive complex 2 (PRC2) and regulates the chromatin state of the *HOXD* cluster (Rinn et al., 2007, Tsai et al., 2010). While different classes of regulatory lncRNAs have been discovered, the functional identity of most lncRNAs remains elusive and some in fact encode small peptides (Martinho et al., 2004, Kondo et al., 2010, Pauli et al., 2014). Understanding the significance of lncRNAs remains an important task facing biology today.

A challenge in identifying lncRNAs is their general lack of sequence conservation across species and many lncRNA genes appear to lack orthologs across different species based on nucleotide sequence similarity. This led to the notion that lncRNA genes do not have the same evolutionary constraints as those of protein-coding genes and the conservation of lncRNAs is inherent in the folded structure (e.g., secondary and tertiary structures), instead of at the primary nucleotide sequence level (Johnsson et al., 2014). Lack of sequence conservation makes it difficult to probe further into the function and evolution of a particular lncRNA gene. Currently, there is no universal experimental approach to characterize the functional contributions of individual lncRNAs, owing to the diversity of functions that are attributed to this class of RNAs.

**Fig. 1:**
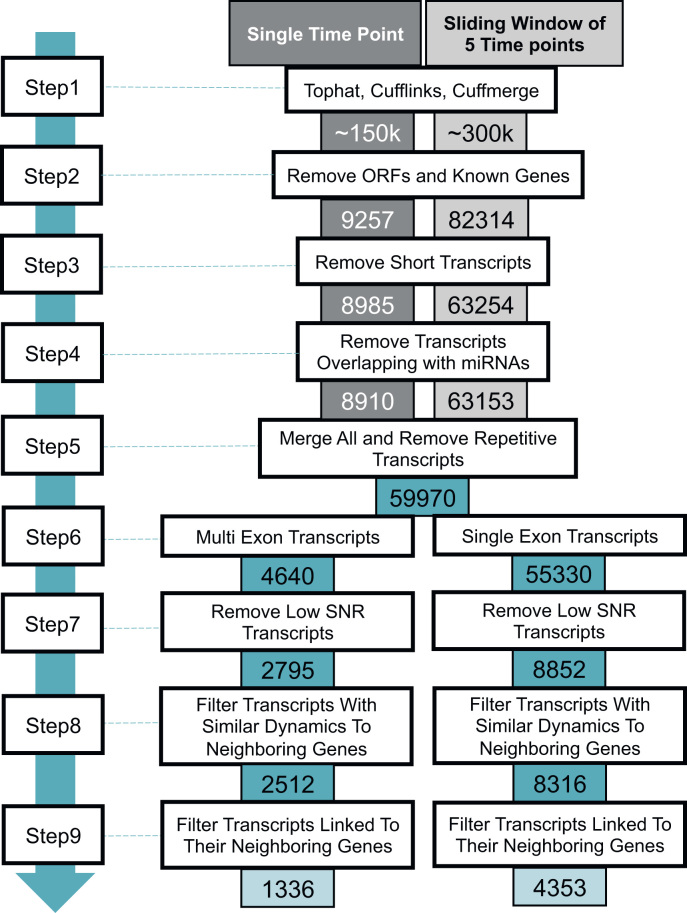
LncRNA discovery pipeline. The output of Cuffmerge (step 1) goes through multiple filtering steps to remove unqualified lncRNA genes and any transcripts with coding potential (step 2), short transcripts (step 3), miRNAs (step 4). These processes are performed in parallel rounds for single time points and also using pooled reads over a sliding window of 5 time points). After these commonly used filtering steps, the remaining transcripts are combined as one set and one representative transcript model is kept among the overlapping transcripts (step 5). Then, multi-exon and single-exon lncRNA candidates are separated (step 6). After removing the lncRNA candidates with less than 5 consecutive time points of non-zero expression, the SNR threshold is applied (step 7). We remove any potential lncRNA candidates that have the possibility of being part of exons of a neighboring gene (step 8 and 9). Our final lists of lncRNAs are 1336 multi-exon lncRNAs and 4353 single-exon lncRNAs.

An effective approach to uncover the function of lncRNAs is to explore temporal and spatial expression profiling. This may be particularly powerful for classes of lncRNAs that have developmentally important roles as expression of such lncRNAs is expected to be both spatially and temporally regulated. Here, we use RNA-seq data from various developmental stages and dissected embryonic tissues and apply a set of search criteria (Fig. 1) to identify both multi-exon and single-exon lncRNAs that have not been described previously. As the first step to systematically identify lncRNAs that are likely to play important developmental functions, we analyzed a set of comprehensive RNA-seq data covering the first 66 h of frog embryogenesis (Owens et al., 2016). In that work, we established a method to quantify the absolute levels of transcripts per embryo and analyzed the temporal expression patterns. Here, we continue with a similar approach employing Gaussian processes, which offer an efficient statistical representation of the high-temporal resolution time-series data analyzed. We make use of Gaussian processes to identify developmentally relevant temporal dynamics of lncRNAs. We have compared the expression trajectories of individual lncRNA genes to the neighboring protein coding genes and identified groups of lncRNAs that show correlative expression profiles with respect to those of the neighboring genes. We propose that this subclass of lncRNAs has *cis*-regulatory functions in development. We also identified lncRNAs showing spatially confined expression patterns in the gastrula stage embryo, implicating their roles during gastrulation.

## 2 Materials and methods

### 2.1 Overview of analysis

We previously obtained high-density RNA-seq time course data covering the first 66 h of *Xenopus tropicalis* development (Owens et al., 2016). Here, RNA-seq reads from polyA+ RNA (GSE65785) were aligned to the *X. tropicalis v9* genome using TopHat (Trapnell, 2009) and Cufflinks (Trapnell, 2010). The initial Cuffmerge output was further subjected to a multi-step filtering process (Fig. 1), after which, we were left with 1336 multi-exon lncRNAs and 4353 single-exon lncRNAs. We modeled the expression dynamics of these lncRNAs using Gaussian processes, which is a commonly used machine learning technique that has been used to model gene expression over time (Gao et al., 2008, Owens et al., 2016).

### 2.2 Xenopus embryo dissection, RNA isolation and sequencing

Synchronously developing *Xenopus tropicalis* embryos were obtained by *in vitro* fertilization using standard methods. Stage 10–10.25 gastrula embryos were manually dissected into five fragments representing ectoderm, dorsal mesoderm, lateral mesoderm, ventral mesoderm and endoderm (Blitz et al., 2016) and RNAs were isolated after homogenization. The RNA samples were subjected to polyA+ selection and library production according to the Illumina Tru-Seq mRNA-seq kit. Libraries were ligated using bar-coded adaptors and subjected to 50-bp single end sequencing on an Illumina HiSeq2000 instrument (Blitz et al., 2016). Dissection RNA-seq datasets can be found in Blitz et al. (2016).

### 2.3 Transcriptome assembly

Our lncRNA detection pipeline started with aligning the timecourse RNA-Seq paired-end reads from each time point (90 samples) to the *Xenopus tropicalis* version 9 genome using TopHat v2.0.12 (Trapnell et al., 2009) and Bowtie2 v2.2.1 (Langmead and Salzberg, 2012). Mapping assignment did not retain the multimapping reads. We constructed the transcripts from mapped reads from each individual time point using Cufflinks v2.2.1 (Trapnell et al., 2010), guided by the *X. tropicalis* version 9 genome. In parallel, we combined the mapped reads from each five consecutive time points (sliding window size of five applied across 90 time course samples) and performed the same analysis. This sliding window approach across the datasets allows us to reliably detect weakly expressed lncRNAs owing to deeper sequence coverage resulting from this compilation. After the assembly step, all of the transcripts were analyzed for possible artifacts and combined into one set using CuffMerge (Trapnell et al., 2010) to create a reference transcriptome. This step was performed separately on transcripts coming from individual time points and also on transcripts generated using the sliding window (step 1 in Fig. 1). These initial sets of transcripts were then subject to multiple filtering steps.

### 2.4 Transcript abundance estimation

To generate the expression profiles of transcripts, we used HTSeq (Anders and Huber, 2010) to count the number of reads mapped to each transcript at each time point, based on Tophat alignment results. These read counts then were normalized by the library size and transcript length and converted to RPKM values.

### 2.5 Gaussian processes to model expression dynamics

Gaussian processes, a machine learning tool used commonly to model biological time series dynamics (Gao et al., 2008, Honkela et al., 2010, Äijö et al., 2014), offer a non-parametric representation of gene expression profiles. Here, we use them to assess the quality of expression dynamics by calculating a signal to noise ratio for each lncRNA candidate. We used Gaussian processes with a Matérn kernel with shape parameter ν=5/2 to model the expression profiles. The Matérn kernel has three hyperparameters: $\sigma_{f}^{2}$ – the signal variance; τ – the timescale (commonly referred to as the lengthscale); and $\sigma_{n}^{2}$ – the noise variance. Roughly, $\sigma_{f}$ measures the scale of the data (the expression level of a given lncRNA); τ – measures how rapidly in time the lncRNAs expression can change; and $\sigma_{n}$ – measures the sample noise around a trend in expression, this is a contributing factor to the width of confidence intervals in Fig. 3. See Owens et al. (2016) for details of the kernel and these hyperparameters. To assess our ability to discern lncRNA dynamics we evaluated the signal-to-noise ratio (SNR) for each lncRNA. This is defined as $\mathrm{SNR}=\log[\sigma_{f}^{2}/\sigma_{n}^{2}]$, and is related to the expression level of a lncRNA divided by the size of the sample noise. Therefore, a larger SNR indicates that the noise is less dominant and that we are better able to characterize the dynamics of the lncRNA (Supplementary Figs. 1 and 2). We use the SNR as an alternative to filtering on expression level alone. As $\sigma_{f}$is correlated to expression level, the SNR is a more informative filter than an expression level filter. Here, Gaussian process analysis is performed using the GPy library in Python (http://sheffieldml.github.io/GPy/).

### 2.6 Strand verification employing strand-specific RNA-seq data

We used available strand-specific data (Collart et al., 2014), covering the first 9 h of our time course, to predict the strand of assembled transcripts. For each transcript, a binomial test was used to find the strand with significantly more mapped reads (p-value: <0.01). These strand predictions were later used to evaluate and modify Cufflinks strand predictions.

### 2.7 Differential spatial expression analysis

To identify differentially expressed transcripts in the early gastrula, HTSeq data was used to find the number of mapped reads on each transcript, for each replicate. These numbers then were analyzed by limma v.3.22.6 (Ritchie et al., 2015), after the RNA-seq data read count were preprocessed by voom transformation (Law et al., 2014). A p-value of 0.05 was used to find significant differentially expressed transcripts.

## 3 Results and discussion

### 3.1 Computational pipeline to identify lncRNAs

Fig. 1 shows the pipeline used to identify lncRNAs in *Xenopus tropicalis* by analyzing an RNA-seq timecourse of closely-spaced timepoints (Owens et al., 2016). We used Cufflinks to discover the transcripts and Cuffmerge to generate a dataset of all detected transcripts. We focused on identifying only intergenic lncRNAs that do not show overlap with coding genes. We examined the coding potential of individual lncRNA transcripts using TransDecoder (https://transdecoder.github.io), and removed the transcripts that have coding potential (step 2). We set a minimum length open reading frame (ORF) to be 100 amino acids (aa) long, which has previously been used to identify lncRNAs (Chen et al., 2016, Clark et al., 2015). Lowering the threshold will exponentially increase the number of ORFs identified (https://github.com/TransDecoder/TransDecoder/), and will lead to the exclusion of many genuine lncRNAs. Next, we removed short transcripts that were less than 200 nucleotides in length (step 3) and that overlapped with miRNAs (step 4). We then combined all the transcripts, checked the overlaps between them, and kept one representative model among overlapping transcripts, resulting in 59,970 lncRNA candidates (step 5). The list resulted in 4640 multi-exon lncRNA candidates and 55,330 single-exon lncRNA candidates (step 6), which were subsequently analyzed separately (steps 7, 8, 9).

#### 3.1.1 Signal to noise ratio

We applied additional filtering steps to remove poor quality lncRNA candidates. We disqualified lncRNA candidates that were not expressed in at least 5 consecutive time points (step 7). This filtering step removed 637 and 32,130 transcripts from the set of multi-exon and single-exon candidates, respectively. Following this, we restricted our attention to those lncRNAs that exhibited consistent dynamics. A simple approach could be to set an absolute expression level threshold and remove low expression lncRNAs. However, this approach may remove lncRNAs that are expressed at consistent, but low levels during development. To avoid the loss of these lncRNAs, we opted for a different approach. We took advantage of our Gaussian process analysis to calculate a signal to noise ratio (SNR) for each gene (the log ratio of signal variance and noise variance hyperparameters, see Methods and Supplementary Figs. 1 and 2 for examples). We set a threshold requiring all lncRNA candidates to have of SNR>0.6 (Supplementary Figs. 1 and 2). After this filtering (step 7), 2795 multi-exon and 8852 single-exon lncRNAs remained.

#### 3.1.2 Strand-assignment

We examined the orientation of the lncRNA transcripts with respect to the closest genes. First, we identified the closest gene on both sides (upstream and downstream) of a candidate lncRNA and determined their relative transcriptional directions (e.g., each lncRNA - neighboring gene pair has a parallel or anti-parallel orientation). Since our RNA-seq data is not strand-specific, making a strand assignment for each lncRNA is challenging. Cufflinks makes a strand prediction based on asymmetric splice junction information in multi-exon lncRNAs. We compared the accuracy of Cufflink's strand prediction for multi-exon lncRNAs to strand calls based on published strand-specific RNA-seq data (Collart et al., 2014). The strand-specific RNA-seq data by Collart et al., are limited to the first 9hours post fertilization, and so this comparison only allows us to evaluate the accuracy of strand calls predicted by Cufflinks over this developmental window. We mapped Collart et al.’s sequencing data to our set of lncRNAs, and applied a binomial test on the number of the mapped reads on each strand to decide the orientation of each candidate. We identified that, of 2795 lncRNA candidates, 1849 multi-exon lncRNA candidates are expressed during the first 9 h of development, and then we validated that 77% (1418 out of 1849 multi-exon lncRNAs) of strand prediction by Cufflinks are accurate. For single-exon lncRNAs, 5108 (58%) of 8852 single-exon lncRNAs are present during the first 9 h of *Xenopus tropicalis* development, and only 53% of these candidates (2707 single-exon lncRNAs) were supported by Collart et al. (2014). Consequently, Cufflinks strand predictions for single-exon genes are no better than random and therefore the strand calls were made based on the Collart et al., data.

Next, we used strand prediction information to further disqualify poor quality lncRNA candidates. We reasoned that if a candidate lncRNA is transcribed as part of an adjacent transcriptional unit (e.g., unrecognized exons), instead of being independently transcribed from a bone fide lncRNA gene, we expect 1) the transcriptional direction of a lncRNA and the neighboring gene to be the same direction, 2) their expression levels to be highly correlated. Pearson correlation coefficients were calculated between each lncRNA and each of the neighboring genes on either side. If Pearson correlation was >0.9 and the lncRNA has the same strand orientation as the correlated gene, then the lncRNA was removed (Step 8). For multi-exon genes, 283 of 2795 were removed. We assigned “class 1” to these if the strand information of the lncRNA was known from Collart et al. (2014), and if the lncRNA and the neighboring genes were transcribed from the same strand; we assigned “class 2” otherwise. Of the 283, 256 were class 1 and 27 were class 2. Similarly, for single-exon lncRNAs, 536 (436 class 1 and 100 class 2) lncRNA candidates were removed.

#### 3.1.3 Paired-end read overlaps

To ensure we identify lncRNAs with high confidence, we have inspected and identified paired-end reads that were mapped on both lncRNA and an adjacent coding gene transcription, as such read pairs are evidence of a physical link between these two transcripts. If a single or multi-mapping paired-end read connects a lncRNA to a neighboring coding gene, then the candidate lncRNAs were removed (step 9), resulting in a final set of 1336 multi-exon lncRNAs and 4353 single-exon lncRNAs. The list of lncRNAs is shown in Supplementary Tables 1–4 (also see https://cbcl.ics.uci.edu/public_data/Xen-LncRNA/). We also include a list of lncRNAs that were removed at step 9 (Supplementary Tables 5 and 6) as this stringent criterion could potentially remove genuine lncRNAs (Supplementary Figs. 3 and 4, see below).

### 3.2 Temporal expression dynamics of lncRNAs

**Fig. 2:**
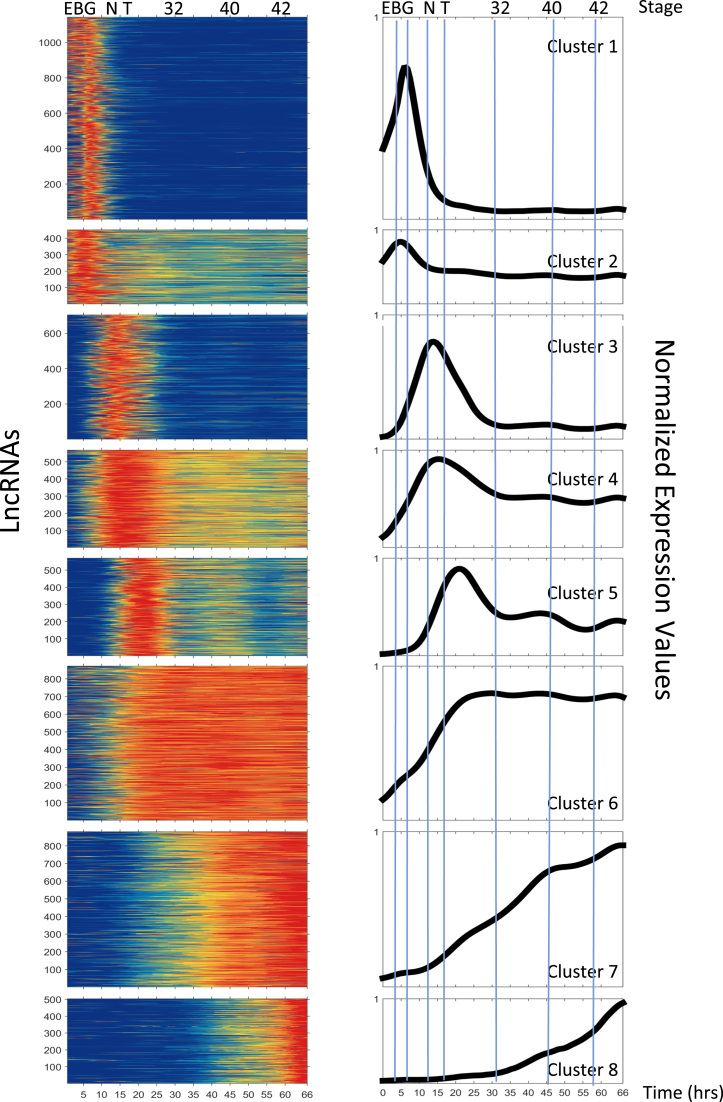
Temporal expression dynamics of lncRNAs. The expression values of individual candidate lncRNAs are normalized by their maxima. These expression profiles are assigned (k-means clustering) to 8 different expression clusters. A) The heatmaps show individual normalized expression patterns for all 5689 lncRNAs. B) The plots demonstrate the average expression of all genes within individual clusters. Each blue bar in panel B corresponds to egg (E), late blastula (B), gastrula (G), neurula (N), tailbud (T).

We examined the temporal expression dynamics of 5689 lncRNAs including both single and multi-exon lncRNAs. We first determined expression values of individual lncRNAs at each time point after normalizing (RPKM) the number of mapped reads based on the length of the lncRNA and the library size. Next, we applied Gaussian processes to generate a smooth representation of the expression profile, and followed this by clustering the expression profiles of the lncRNAs based on k-means clustering (Fig. 2). The results show that lncRNA expression is dynamically regulated during the course of embryonic development. Cluster 1 lncRNAs are maternally expressed and drop to very low levels shortly after zygotic transcription initiates. Cluster 2 lncRNAs are also maternally expressed, but the expression persists through the timecourse. Cluster 3, 4 and 5 lncRNAs are zygotically activated with increasingly later expression peaks and differing dynamics of later temporal expression. Cluster 6, 7 and 8 lncRNAs are zygotically activated, but their expression persists for a prolonged period of time. The time course analysis indicates that many lncRNAs are developmentally regulated and thus likely to have developmentally relevant functions.

### 3.3 Expression correlation between lncRNAs and neighboring genes

**Fig. 3:**
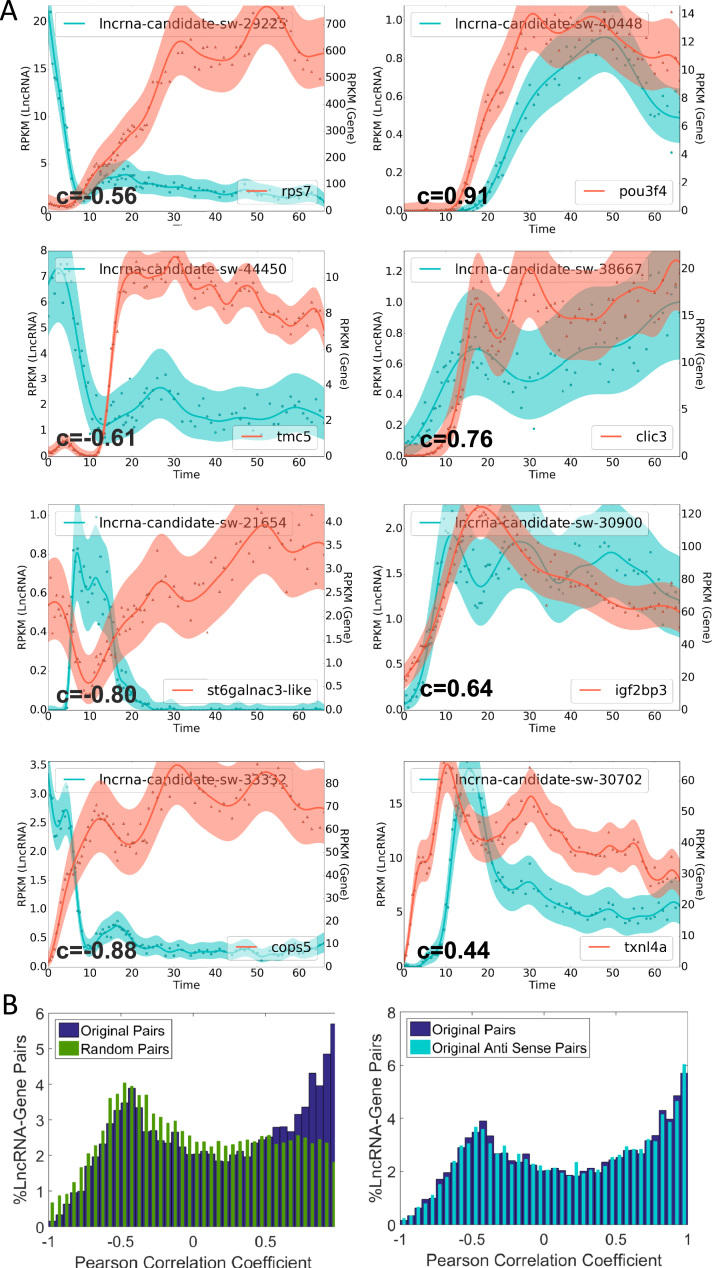
Expression profiles of lncRNAs and the neighboring genes. A) Gene expression values in RPKM are shown for a lncRNA and a neighboring gene during the developmental time course. The blue and red solid lines represent Gaussian processes medians and the shaded areas are the 95% confidence intervals of the data. C denotes the Pearson correlation between the lncRNA and neighboring gene expression dynamics. Gene models of lncRNAs are shown in Supplementary Fig. 5. B) Left panel shows distribution of correlations of pairs of lncRNA – neighboring gene (in blue) and pairs of lncRNA – random gene (green). Right panel shows the distribution of correlations of pairs of lncRNA – neighboring gene (in blue) and pairs of antisense strand lncRNA –neighboring gene (light blue). Pearson coefficient of 1 is highly correlated, and −1 is highly anti-correlated.

It has been shown that lncRNAs are often located in close proximity to coding genes (Sigova et al., 2013, Rinn and Chang, 2012). We investigated the correlation in expression between lncRNAs and neighboring genes. The motivation behind this approach is to identify lncRNA genes that may act locally to affect neighboring gene expression, or vice versa. We calculated the Pearson correlation coefficient for lncRNA and smoothened gene expression profiles. Fig. 3A shows representative examples of the correlation of eight lncRNA – neighboring gene pairs. Supplementary Fig. 5 shows gene browser views illustrating the genomic positional relationships between these eight lncRNA and neighboring gene pairs. We have performed permutation analysis to determine whether adjacent lncRNA-gene pairs have greater correlation than expected at random. Fig. 3B (left panel) shows that adjacent lncRNA-neighboring gene pairs are more correlated than lncRNA-random gene pairs. In order to ensure that the correlation observed is not due to some lncRNAs in our set that are actually part of neighboring genes, we selected lncRNA-gene pairs on opposing strand (the direction of lncRNA transcription and adjacent genes are opposite), and examined the relationship between strand and correlation. We found that opposing strand lncRNAs-adjacent gene pairs correlated well (Fig. 3B, right panel), thus suggesting that these lncRNAs have cis-regulatory roles. We also note that a significant number of lncRNA-neighboring gene pairs show no correlation, which indicates that these lncRNAs may have novel biological functions.

### 3.4 Spatially regulated lncRNA expression

**Fig. 4:**
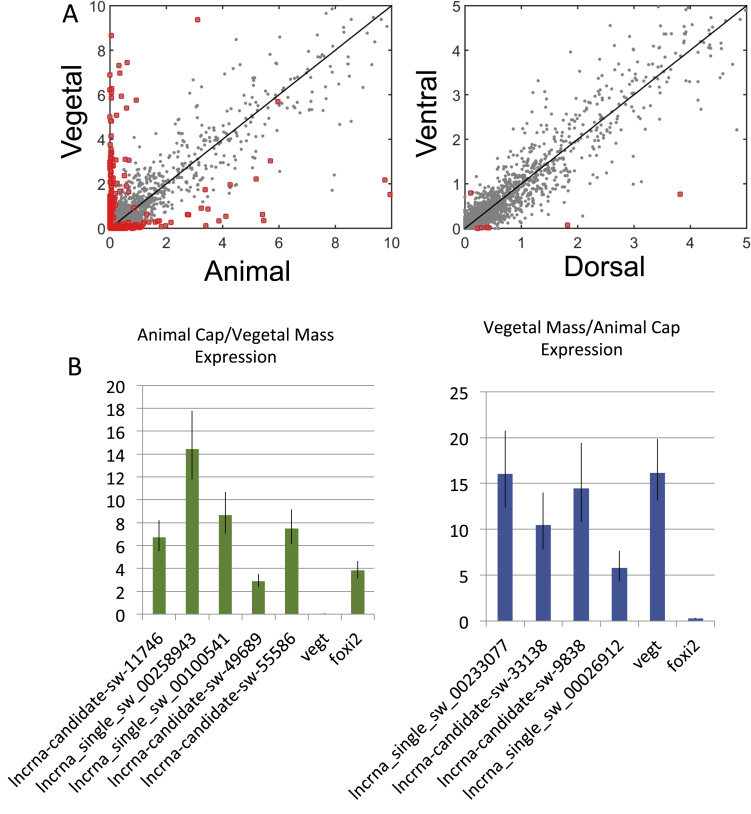
LncRNA distribution in gastrula stage embryos. A) Spatial expression of lncRNAs in gastrula stage embryos. The scatter plot in left panel depicts the comparison between vegetal and animal RPKM values of lncRNAs. The scatter plot in the right panel depicts the comparison between ventral and dorsal expressions. Individual points represent 5689 lncRNAs expressed in gastrula embryos, and the red boxes mark differentially expressed lncRNAs. The black line denotes equal expression between vegetal and animal, or dorsal and ventral tissue fragements. B) RT-qPCR analysis of lncRNAs using RNA isolated from designated tissue fragements.

Previous studies have reported that lncRNA expression can be cell-type or tissue-type specific and may vary spatially across different tissues (Derrien et al., 2012). We obtained RNA-seq data from dissected embryonic tissues (animal poles, dorsal, lateral and ventral marginal zones, and vegetal masses, representing ectoderm, dorsal mesoderm, lateral mesoderm, ventral mesoderm and endoderm, respectively) at the gastrula stage (Blitz et al., 2016). Reads from these datasets were mapped to our lncRNA collection identified in this study. Using the software limma (Ritchie et al., 2015), we compared animally (ectoderm) and vegetally (endoderm) enriched lncRNAs. Of the 4353 single-exon and 1336 multi-exon lncRNAs, we find 266 single-exon lncRNAs and 65 multi-exon lncRNAs that are expressed in a spatially defined manner (Fig. 4, Supplementary Table 7). When a similar analysis was performed for dorsally or ventrally (mesoderm) enriched lncRNAs, we only identified 8 lncRNAs (5 single-exon and 3 multi-exon) (Supplementary Table 8). We have independently validated the localization data by performing RT-qPCR analysis of RNA samples from dissected tissue fragments using specific primers (Fig. 4B, Supplementary Table 9), which confirmed the results of RNA-seq. We propose that these lncRNAs may be involved in regulating the expression of germ layer-specific genes.

### 3.5 Accounting for lncRNA model inaccuracies

Identification of lncRNAs in the genome is challenging because they are not well conserved at the primary sequence level. In addition, some lncRNAs are transcribed from intronic regions of genes, while others are transcribed from intergenic regions. For identification of lncRNAs near coding genes, stranded RNA-seq data is key to understand the transcriptional architecture. For example, it simplifies the discrimination between a genuine lncRNA and as yet unannotated exon of a coding gene. Our timecourse RNA-seq (Owens et al., 2016) is not strand-specific data, and whilst we have taken a conservative strategy to identify lncRNAs, strand-specific data will nevertheless be beneficial. In our list of lncRNAs (Supplementary Tables 1, 2, 5 and 6), whenever the strand of lncRNAs is confirmed with respect to the neighboring genes, it is indicated. Our current analysis should provide a comprehensive and useful list for further study. Our stringent criteria have removed any lncRNAs, for example, with very high temporal correlations to neighboring genes with the same strandedness, or with a single paired-end read connecting a lncRNA to a neighboring gene. This criterion may be overly stringent and, thus, we may have discarded genuine lncRNAs from our current list. Examples of such include lncRNAs associated with *foxa2* and *sox2* (Supplementary Fig. 3), and evolutionarily conserved lncRNAs such as the *malat1/neat2* lncRNAs. All these lncRNAs were discarded because one paired-end read bridged lncRNA and adjacent gene exon. However, these lncRNAs are conserved in human and mouse. In addition, we note that evolutionarily conserved *Xlsirts-*like lncRNAs have survived our pipeline analysis (Supplementary Fig. 4).

## 4 Concluding remarks

Two main challenges exist in uncovering the function of lncRNAs. First is the identification of bona-fide lncRNAs and the second is to infer biological functions of these lncRNAs. In this study, we described a systematic pipeline to identify *Xenopus tropicalis* lncRNAs during early embryonic development. We demonstrate the usefulness of applying Gaussian processes to identify dynamic expression patterns of lncRNAs that may be involved in developmental roles. With available RNA-seq data and bioinformatics tools, we identified thousands of multi-exon and single-exon lncRNAs that show interesting temporal expression dynamics. The next step is to reveal their precise biological mechanisms and the links to pathogenesis in various diseases. The *Xenopus* system is likely to contribute significantly to the understanding of lncRNA biology because the system is ideally suited to perform experimental embryology, ectopic/overexpression and genome editing in whole animals.

## Appendix A Supplementary material

Supplementary Table 1: List of multi-exon lncRNA (1336 lncRNAs after step 9)

.

Supplementary Table 2: List of single-exon lncRNA (4353 lncRNAs after step 9)

.

Supplementary Table 3: multi exon lncRNA sequences

.

Supplementary Table 4: single exon lncRNA sequences

.

Supplementary Table 5: Additional list of multi-exon lncRNA (1184 lncRNAs)

.

Supplementary Table 6: Additional list of single-exon lncRNA (3963 lncRNAs)

.

Supplementary Table 7: lncRNAs differentially localized animally and vegetally

.

Supplementary Table 8: lncRNAs differentially localized dorsally and vegetally

.

Supplementary Table 9: RT-qPCR Primer pairs

.

Supplementary Figure 1 Scatter plot of 4640 lncRNA candidates after step 6. X-axis is the log (signal variance/noise variance) calculated using signal variance and noise variance hyperparameters of Gaussian processes. Y-axis is the log of maximum expression value observed for each transcript. The light purple points (2795) have at least 5 consecutive non-zero expression time points and those in blue (1845) failed to satisfy this condition. The vertical line demonstrates log10(SNR)=0.6, which is the threshold we used to eliminate less-qualified transcripts. Supplementary Figure 2 Examples of lncRNA gene expression patterns at increasing signals-to-noise ratios (SNRs). We set the threshold SNR to be 0.6 for our pipeline analysis (Figure 1). Supplementary Figure 3 Candidate lncRNAs located near *foxa2* and *sox2* loci. Foxa2 lncRNA did not survive our analysis as one paired end read bridged between this lncRNA and Foxa2 exon. However, we propose that this lncRNA adjacent to *FOXA2* is an authentic lncRNA because syntenic lncRNA is found in both human and mouse. We also found lncRNAs adjacent to Sox2 in both human and mouse.Supplementary Figure 4*MALAT/NEAT2* and *Xlsirts*-related lncRNAs in *Xenopus tropicalis.* A) Besides the sequence similarity between lncrna_single_sw_00112474 and *MALAT1* in mouse and human, the relative position of frog *malat1* with respect to *frmd8* also agrees with that found in mouse and human. In all three species, *MALAT1* is a single exon lncRNA gene located downstream of *FRMD8*. In addition, *NEAT1* lncRNA gene is located between *MALAT1* and *frmd8* in human and mouse, and we find a lncRNA in the similar position in *Xenopus tropicalis*. B) For *Xlsirts*, multiple alignments to our set of lncRNAs were reported by Blast, and two examples are shown*. Xlsirt-*related genes are found between *Xenopus tropicalis* and *laevis,* but not in human and mouse. Supplementary Figure 5 Genome browser view of lncRNAs and neighboring genes displayed in Figure 3. Red lines represent coding genes. Blue lines denote lncRNAs. Grey lines feature other nearby coding genes around the lncRNA loci.
