# Supplementary material for: Developmentally regulated long non-coding RNAs in Xenopus tropicalis
Source: Dev Biol. 2017 Jun 15;426(2):401–8. doi: 10.1016/j.ydbio.2016.06.016 (PMC5233649; doi:10.1016/j.ydbio.2016.06.016)
Supplement: Supplementary file 10 — Supplementary Figure 1 Scatter plot of 4640 lncRNA candidates after step 6. X-axis is the log (signal variance/noise variance) calculated using signal variance and noise variance hyperparameters of Gaussian processes. Y-axis is the log of maximum expression value observed for each transcript. The light purple points (2795) have at least 5 consecutive non-zero expression time points and those in blue (1845) failed to satisfy this condition. The vertical line demonstrates log10(SNR)=0.6, which is the threshold we used to eliminate less-qualified transcripts. Supplementary Figure 2 Examples of lncRNA gene expression patterns at increasing signals-to-noise ratios (SNRs). We set the threshold SNR to be 0.6 for our pipeline analysis (Figure 1). Supplementary Figure 3 Candidate lncRNAs located near foxa2 and sox2 loci. Foxa2 lncRNA did not survive our analysis as one paired end read bridged between this lncRNA and Foxa2 exon. However, we propose that this lncRNA adjacent to FOXA2 is an authentic lncRNA because syntenic lncRNA is found in both human and mouse. We also found lncRNAs adjacent to Sox2 in both human and mouse.Supplementary Figure 4MALAT/NEAT2 and Xlsirts-related lncRNAs in Xenopus tropicalis. A) Besides the sequence similarity between lncrna_single_sw_00112474 and MALAT1 in mouse and human, the relative position of frog malat1 with respect to frmd8 also agrees with that found in mouse and human. In all three species, MALAT1 is a single exon lncRNA gene located downstream of FRMD8. In addition, NEAT1 lncRNA gene is located between MALAT1 and frmd8 in human and mouse, and we find a lncRNA in the similar position in Xenopus tropicalis. B) For Xlsirts, multiple alignments to our set of lncRNAs were reported by Blast, and two examples are shown. Xlsirt-related genes are found between Xenopus tropicalis and laevis, but not in human and mouse. Supplementary Figure 5 Genome browser view of lncRNAs and neighboring genes displayed in Figure 3. Red lines represent coding [file mmc10.pptx]

## Slide 1
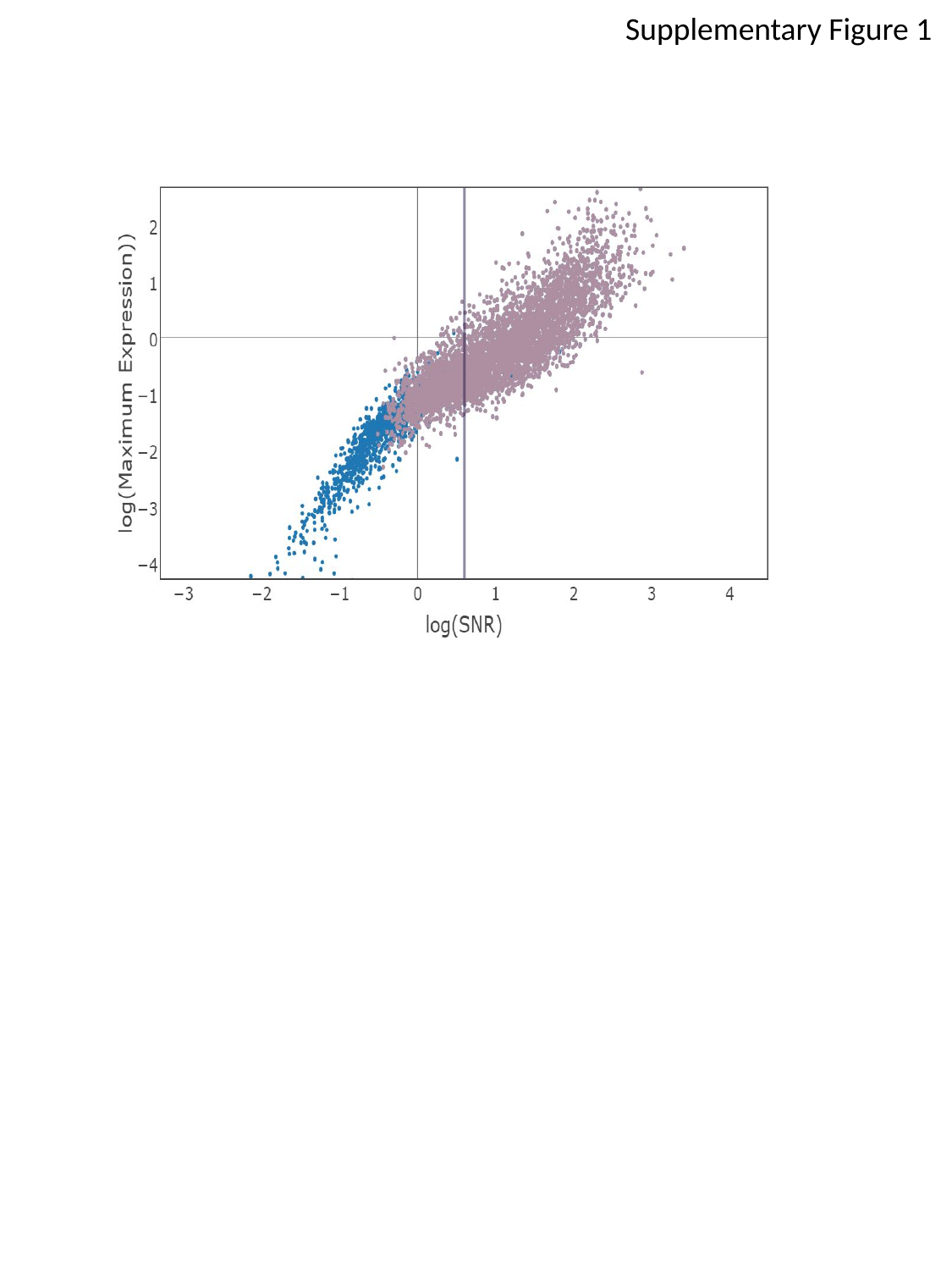

Supplementary Figure 1

## Slide 2
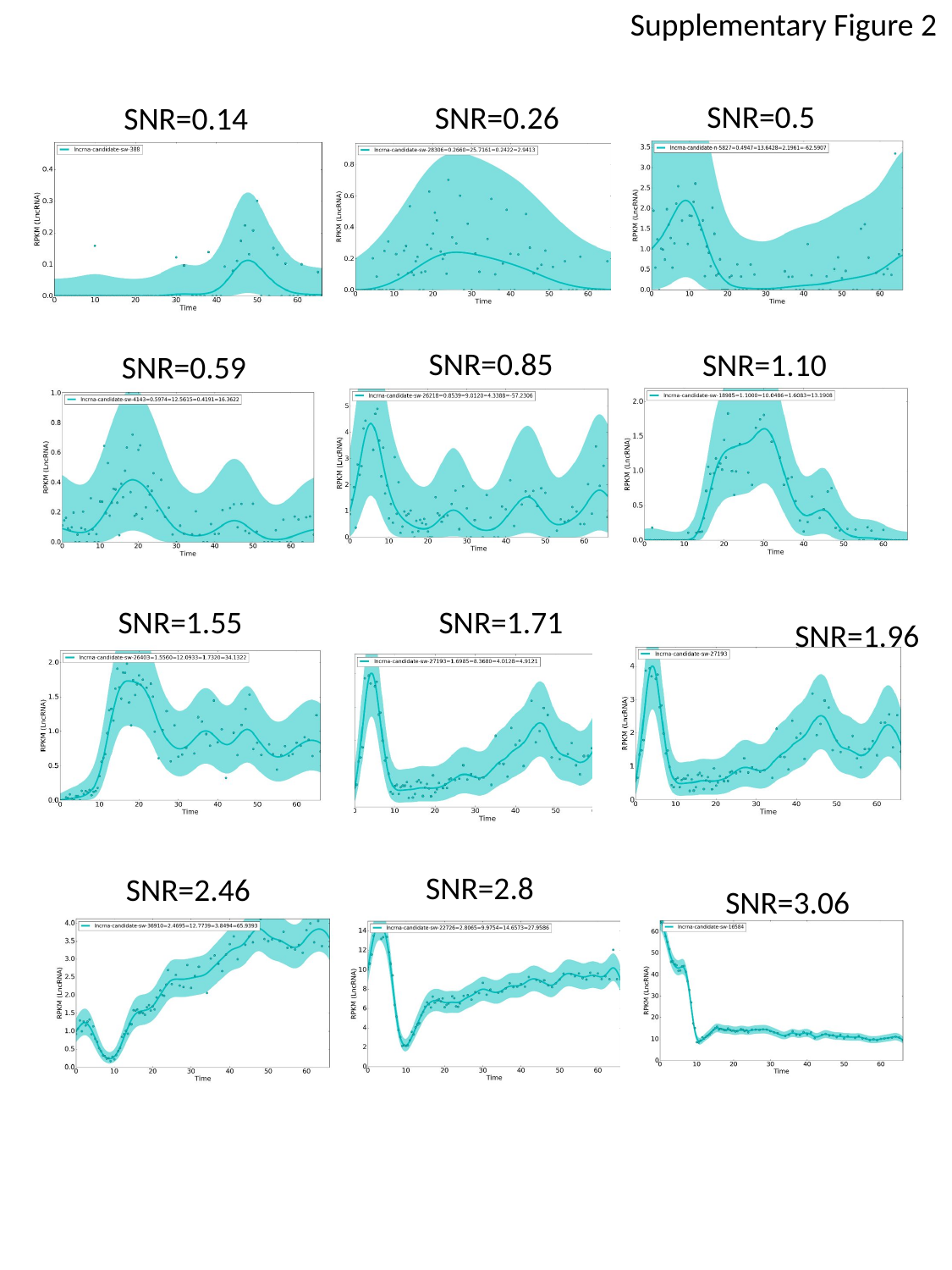

Supplementary Figure 2
SNR=0.5
SNR=0.26
SNR=0.14
 SNR=0.85
SNR=1.10
SNR=0.59
SNR=1.71
SNR=1.55
SNR=1.96
SNR=2.8
SNR=2.46
SNR=3.06

## Slide 3
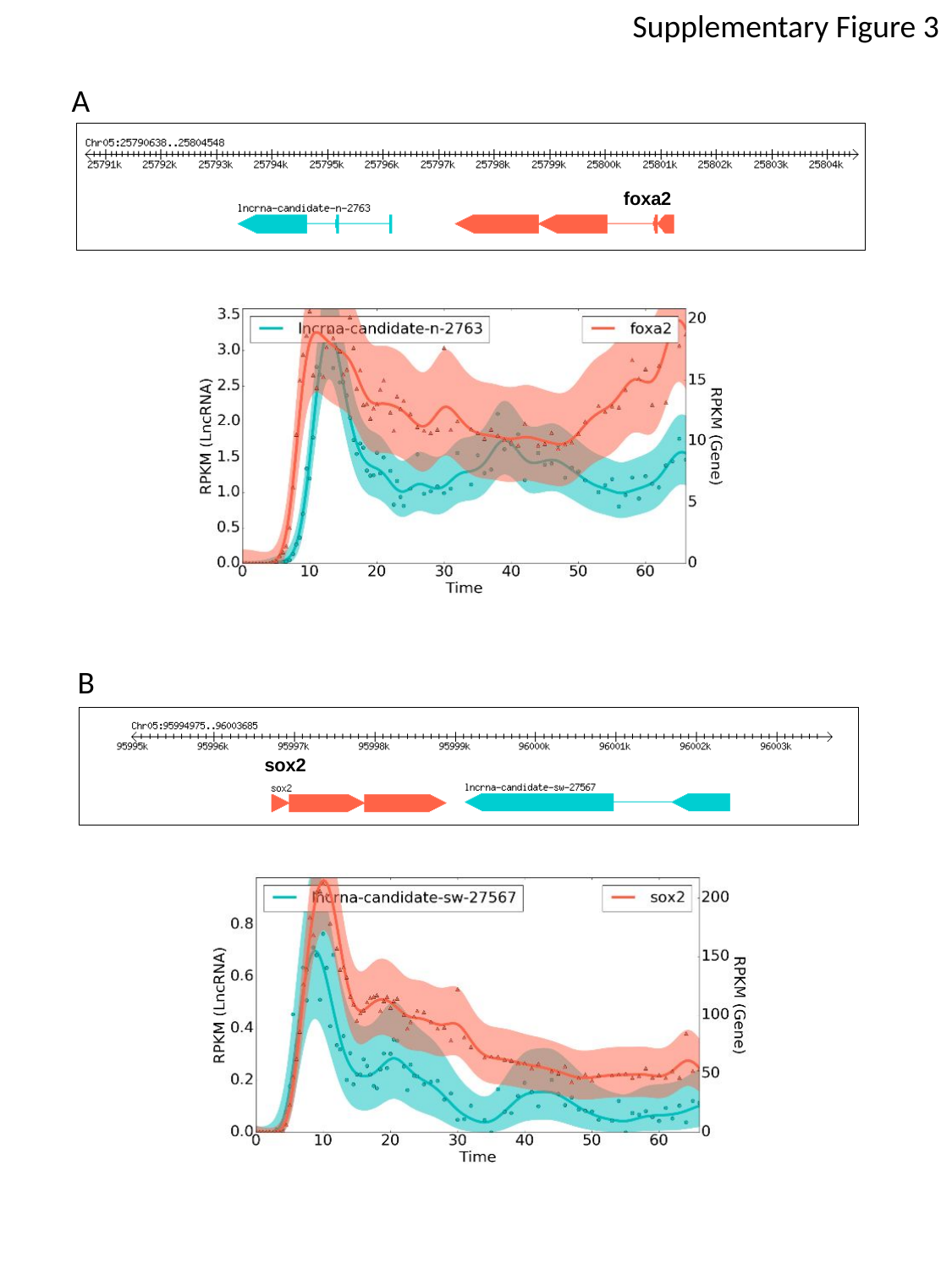

Supplementary Figure 3
A
foxa2
B
sox2

## Slide 4
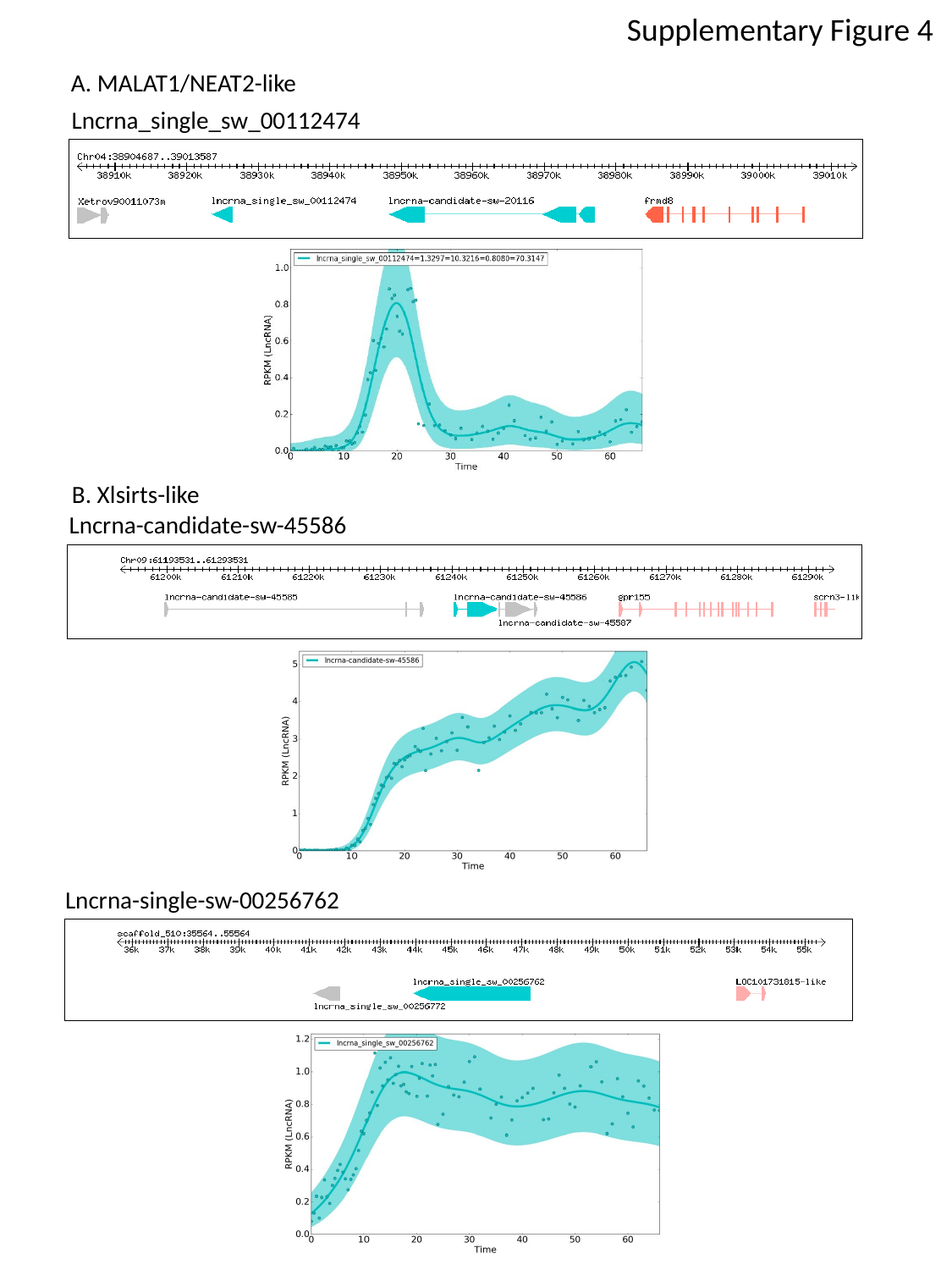

Supplementary Figure 4
A. MALAT1/NEAT2-like
Lncrna_single_sw_00112474
B. Xlsirts-like
Lncrna-candidate-sw-45586
Lncrna-single-sw-00256762

## Slide 5
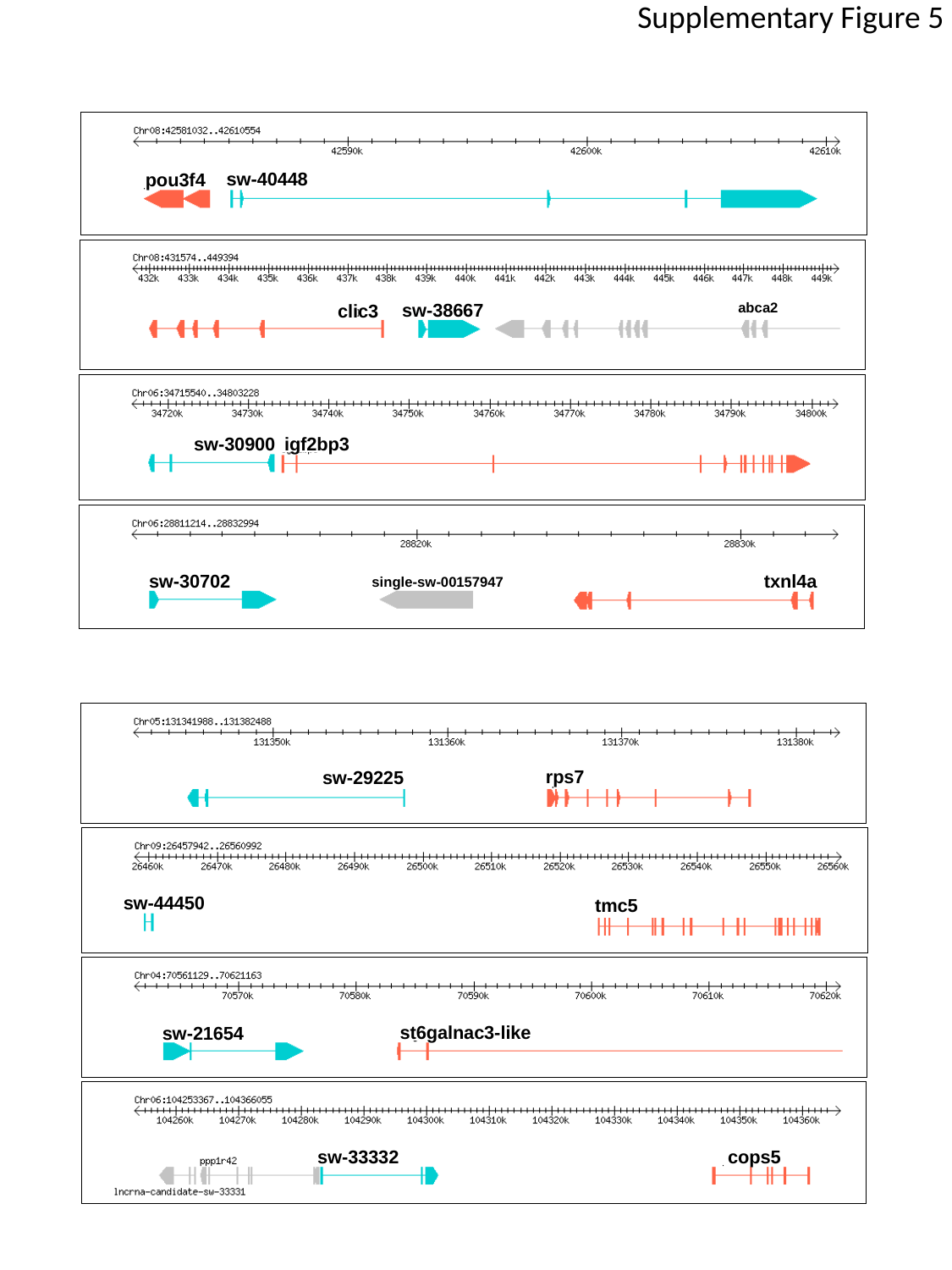

Supplementary Figure 5
sw-40448
pou3f4
sw-38667
abca2
clic3
sw-30900
igf2bp3
sw-30702
txnl4a
single-sw-00157947
rps7
sw-29225
sw-44450
tmc5
st6galnac3-like
sw-21654
sw-33332
cops5
